# US Consumers’ Understanding of Nutrition Labels in 2013: The Importance of Health Literacy

**DOI:** 10.5888/pcd14.170066

**Published:** 2017-09-28

**Authors:** Alexander Persoskie, Erin Hennessy, Wendy L. Nelson

**Affiliations:** 1 Office of Science, Center for Tobacco Products, US Food and Drug Administration, Silver Spring, Maryland; 2 Tufts University, Friedman School of Nutrition Science and Policy, Boston, Massachusetts; 3 Behavioral Research Program, National Cancer Institute, National Institutes of Health, Bethesda, Maryland

## Abstract

**Introduction:**

We examined US adults’ understanding of a Nutrition Facts panel (NFP), which requires health literacy (ie, prose, document, and quantitative literacy skills), and the association between label understanding and dietary behavior.

**Methods:**

Data were from the Health Information National Trends Survey, a nationally representative survey of health information seeking among US adults (N = 3,185) conducted from September 6, 2013, through December 30, 2013. Participants viewed an ice cream nutrition label and answered 4 questions that tested their ability to apply basic arithmetic and understanding of percentages to interpret the label. Participants reported their intake of sugar-sweetened soda, fruits, and vegetables. Regression analyses tested associations among label understanding, demographic characteristics, and self-reported dietary behaviors.

**Results:**

Approximately 24% of people could not determine the calorie content of the full ice-cream container, 21% could not estimate the number of servings equal to 60 g of carbohydrates, 42% could not estimate the effect on daily calorie intake of foregoing 1 serving, and 41% could not calculate the percentage daily value of calories in a single serving. Higher scores for label understanding were associated with consuming more vegetables and less sugar-sweetened soda, although only the association with soda consumption remained significant after adjusting for demographic factors.

**Conclusion:**

Many consumers have difficulty interpreting nutrition labels, and label understanding correlates with self-reported dietary behaviors. The 2016 revised NFP labels may address some deficits in consumer understanding by eliminating the need to perform certain calculations (eg, total calories per package). However, some tasks still require the ability to perform calculations (eg, percentage daily value of calories). Schools have a role in teaching skills, such as mathematics, needed for nutrition label understanding.

## Introduction

The Nutrition Facts panel (NFP) — sometimes referred to as the “cornerstone of nutrition labeling” (1) — was developed to provide US consumers with the information they need to follow dietary recommendations. The Nutrition Labeling and Education Act of 1990 (2), which required nearly all packaged foods to carry the NFP label, was intended to allow consumers to make healthy food choices and ultimately reduce their risk of illness and death from diet-related chronic diseases (3). Many consumers check food labels when buying food, either to choose healthy foods or to lose weight (4). Label users also tend to report more healthful dietary practices than nonusers (5). However, mandated nutrition labels have been criticized for being too complex for many consumers to understand and use (4,6). Comprehension may be particularly difficult for certain subsets of the population, such as consumers with low levels of literacy and numeracy. This is particularly important given the rising rate of obesity and associated medical conditions in the United States.

Understanding the NFP label requires health literacy, that is, “the capacity to obtain, process, and understand basic health information and services needed to make appropriate health decisions” (7). However, a sizable proportion of the US population is deficient in health literacy. The 2003 National Assessment of Adult Literacy (8) found that more than one-third of the US population had only basic or below-basic health literacy, meaning they would have difficulty viewing the nutrition labels of 2 different potato chip packages and determining the difference in the number of calories. Some studies have found that even high school graduates and college students lack the basic health literacy skills to effectively apply nutrition label information (9,10). Beyond education level, little empirical evidence is available about other demographic and behavioral characteristics that may be associated with the ability to interpret information on a nutrition label. Moreover, much of what we know about US consumers’ understanding and use of nutrition labels has come from studies that have relied on convenience samples of participants from shopping malls, grocery stores, and patient populations, rather than nationally representative surveys (6,10–12).

Inadequate health literacy has been associated with many adverse health outcomes, including poor health status, poor health management, more frequent emergency department visits, and unhealthy diets (13,14). Recognizing the importance of health literacy, the Newest Vital Sign (NVS) instrument (15) was developed as a rapid health literacy screening tool that could be readily implemented in clinical practice. To complete the NVS, a person reads an ice cream nutrition label and answers 6 questions that require prose literacy (the ability to search and understand information on a label), document literacy (the ability to find and use information in charts and tables), and quantitative literacy (the ability to perform computations and use numbers embedded in printed materials) (8). The NVS has been validated and used successfully in various clinical settings and patient populations (16–19).

This study examined how a nationally representative sample of the US population performed on a shortened version of the NVS. We examined whether people could use the NFP label on an ice cream container to understand food content (eg, number of calories and grams of carbohydrate per serving) and the effects of a change in consumption on calorie or nutrient intake. The study also examined associations between NFP label understanding and dietary behaviors, including consumption of sugar-sweetened soda, fruits, and vegetables. We reasoned that if the NFP label promotes healthy eating, then people who are better at understanding nutrition labels may be more likely to eat healthier diets, including consuming less sugar-sweetened soda. Moreover, given that the NVS is used to screen for health literacy, we also expected performance to correlate with healthier dietary behaviors, including higher consumption of fruits and vegetables.

## Methods

### Study design

Data were obtained from the National Cancer Institute’s Health Information National Trends Survey (HINTS) Round 4 Cycle 3, a national survey of the US adult, civilian noninstitutionalized population that monitors health information use and cancer-related behaviors. The survey is available in English and Spanish. Details of survey development and methods have been published (20) and are available online (http://hints.cancer.gov). Data were collected by mailed questionnaire from September 6, 2013, through December 30, 2013. Households were randomly selected using a stratified sample from a listing of all nonvacant residential addresses in the United States. One adult per household was asked to complete the questionnaire (ie, the individual with the next birthday). A total of 3,185 surveys were completed, yielding a response rate of 35.2%. HINTS Round 4 Cycle 3 was approved by the Westat Institutional Review Board and was deemed exempt from institutional review board review by the National Institutes of Health’s Office of Human Subjects Research.

### Measures

**NFP label questions.** Participants read an NFP label from an ice cream container (Figure) and answered 4 questions from the NVS instrument, a screening test for health literacy that assesses the ability to understand information contained on a nutrition label (15). Participants responded to the following open-ended questions: “If you eat the entire container, how many calories will you eat?” (item 1, Total Calories); “If you are allowed to eat 60 g of carbohydrates as a snack, how much ice cream could you have?” (item 2, Nutrient Specific); “Your doctor advises you to reduce the amount of saturated fat in your diet. You usually have 42 g of saturated fat each day, which includes 1 serving of ice cream. If you stop eating ice cream, how many grams of saturated fat would you be consuming each day?” (item 3, Health Recommendation); and “If you usually eat 2,500 calories in a day, what percentage of your daily value of calories will you be eating if you eat 1 serving?” (item 4, Daily Value). The study excluded 2 questions from the NVS because of space limitations on the study instrument. Those questions ask if it would be safe to eat the ice cream if you are allergic to penicillin, peanuts, latex gloves, and bee stings, and if not, why not.

**Figure:**
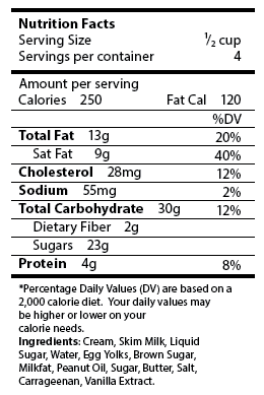
Health Information National Trends Survey (HINTS) Nutrition Facts panel. Copyright Pfizer Inc. All rights reserved.

**Dietary behaviors**. Participants reported the number of days they drank sugar-sweetened soda or pop each week: “Not counting any diet soda or pop, about how often do you drink regular soda or pop in a typical week?” Response options were “every day,” “5 or 6 days a week,” “3 or 4 days a week,” “1 or 2 days a week,” “less often than 1 day a week,” and “I don’t drink any regular soda or pop.” Participants estimated their daily vegetable consumption: “About how many cups of vegetables (including 100% pure vegetable juice) do you eat or drink each day?” Response options were “none,” “ ½ cup or less,” “½ cup to 1 cup,” “1 to 2 cups,” “2 to 3 cups,” “3 to 4 cups,” and “4 or more cups.” Finally, participants estimated their daily fruit consumption using the same response options. To help estimate their daily intake of vegetables and fruit, participants were provided with lists of items equivalent to 1 cup of vegetables (eg, 3 broccoli spears, 12 baby carrots, 1 large ear of corn) and 1 cup of fruit (eg, 1 small apple, 1 medium pear, 1-inch–thick wedge of watermelon).

**Demographic factors.** Participants reported their sex (female; male), age (18–34 y; 35–49 y; 50–64 y; 65–74 y; ≥75 y), highest level of educational attainment (less than a high school diploma; high school diploma; some college; 4-year college degree), household income (<$20,000; $20,000 to <$35,000; $35,000 to <$50,000; $50,000 to <$75,000; ≥$75,000), and race/ethnicity (Hispanic, non-Hispanic white, non-Hispanic black, other).

### Data analysis

The 4 NFP label questions were scored as either correct or incorrect/missing (dichotomous outcome) and summed to create a total performance score (continuous outcome). Twenty participants were excluded from the analysis, because they were administered a short form of the survey that did not include the NFP label items, leaving a total sample of 3,165.

Descriptive analyses and simple linear regressions evaluated differences in NFP label item scores across demographic groups (sex, education, income, age, race/ethnicity). Ordinal logistic regressions evaluated the association between NFP label scores and consumption of fruits, vegetables, and sugar-sweetened soda. The latter regressions were conducted with and without adjusting for demographic characteristics. List-wise deletion was used for each analysis (ie, participants were excluded from any analysis in which there were missing data on one or more of the variables). Alpha was set at .05. Analyses were weighted to account for the survey’s complex sampling design and to generate nationally representative estimates using SAS-Callable SUDAAN version 11.0.0 (RTI International).

## Results

### NFP label understanding

We summarized the demographic characteristics of the sample and the bivariate associations between demographics and NFP label understanding (Tables 1 and 2). We found a strong bivariate association between income and NFP label understanding: participants in the lowest income group correctly answered, on average, 1.31 fewer questions than those in the highest income group. Race/ethnicity was also associated with NFP label understanding: average scores tended to be lower among blacks and Hispanics than among non-Hispanic whites. Age was negatively associated with NFP label understanding: participants in the oldest age group scored lower than those in all other age groups. NFP label understanding was strongly associated with education: on average, participants with less than a high school diploma correctly answered 1.37 of the 4 NFP label questions, participants with a high school diploma correctly answered an average of 2.49 questions, and participants with at least some college correctly answered an average of 3.15 questions. More than one-third (35.4%) of participants with less than a high school diploma were unable to correctly answer any of the NFP label questions, and fewer than 9% could answer all 4 questions correctly. Only 54% of participants with a 4-year college degree correctly answered all 4 nutrition label questions (Table 2).

**Table 1:** Nutrition Facts Panel Scores,^a^ by Demographic Characteristics, Health Information National Trends Survey, United States, 2013

Characteristic	Demographic Predictors of Nutrition Label Score			
	Unweighted No. (Weighted %)	Nutrition Label Score, Mean (95% Confidence Interval)^a^	*B* (Standard Error)^b^	*P* Value
**Sex**				
Female	1,893 (51.7)	2.73 (2.65–2.82)	[Reference]	—
Male	1,190 (48.3)	2.77 (2.66–2.89)	0.04 (0.07)	.59
**Education**				
Less than high school diploma	291 (9.6)	1.37 (1.16–1.59)	−1.78 (0.12)	<.001
High school diploma	932 (33.2)	2.49 (2.36–2.62)	−0.66 (0.09)	<.001
Some college	690 (23.9)	3.15 (3.04–3.25)	−0.01 (0.08)	.94
4-Year college degree	1,164 (33.3)	3.15 (3.04–3.27)	[Reference]	—
**Income, $**				
<20,000	670 (20.1)	1.97 (1.74–2.20)	−1.31 (0.12)	<.001
20,000–<35,000	411 (14.3)	2.58 (2.41–2.76)	−0.70 (0.10)	<.001
35,000–<50,000	393 (14.7)	3.00 (2.81–3.19)	−0.28 (0.12)	.03
50,000–<75,000	446 (17.8)	2.91 (2.75–3.08)	−0.37 (0.09)	<.001
≥75,000	801 (33.1)	3.28 (3.15–3.41)	[Reference]	—
**Age, y**				
18–34	425 (27.2)	3.00 (2.84–3.16)	1.04 (0.11)	<.001
35–49	703 (29.4)	2.78 (2.64–2.93)	0.83 (0.12)	<.001
50–64	1,065 (24.4)	2.85 (2.73–2.97)	0.89 (0.12)	<.001
65–74	510 (9.1)	2.54 (2.39–2.69)	0.58 (0.12)	<.001
≥75	359 (7.7)	1.96 (1.76–2.15)	[Reference]	—
**Race/ethnicity**				
Non-Hispanic white	1,584 (67.2)	3.17 (3.09–3.24)	[Reference]	—
Non-Hispanic black	421 (10.5)	2.00 (1.72–2.27)	−1.17 (0.14)	<.001
Hispanic	494 (15.0)	2.17 (2.00–2.34)	−1.00 (0.09)	<.001
Other	209 (7.3)	2.71 (2.40–3.02)	−0.46 (0.16)	.005

Abbreviation: —, not applicable.

a Nutrition label scores were the total number of correct answers out of 4 Nutrition Facts panel questions (range: 0–4). Scores were based on answers to the following questions: Question 1: “If you eat the entire container, how many calories will you eat?”; question 2, “If you are allowed to eat 60 g of carbohydrates as a snack, how much ice cream could you have?”; question 3, “Your doctor advises you to reduce the amount of saturated fat in your diet. You usually have 42 g of saturated fat each day, which includes 1 serving of ice cream. If you stop eating ice cream, how many grams of saturated fat would you be consuming each day?”; question 4, “If you usually eat 2,500 calories in a day, what percentage of your daily value of calories will you be eating if you eat one serving?” Unweighted frequencies do not total to 3,165 because of missing data on demographic variables.

b *B* = unstandardized regression coefficients from weighted simple linear regressions of nutrition label scores on demographic variables (each demographic variable entered separately).

**Table 2:** Nutrition Facts Panel Scores,^a^ by Educational Level, Health Information National Trends Survey, United States, 2013

Nutrition Label Score	Education Level, Unweighted No. (Weighted %)			
	Less than High School Diploma (n = 291)	High School Diploma (n = 932)	Some College (n = 690)	4-Year College Degree (n = 1,164)
**No. of correct answers**				
0	121 (35.4)	186 (12.7)	86 (4.6)	65 (5.5)
1	68 (23.0)	147 (12.7)	74 (5.6)	78 (5.5)
2	46 (19.1)	167 (18.5)	103 (10.2)	129 (11.0)
3	33 (13.8)	208 (25.2)	181 (30.0)	265 (24.0)
4	23 (8.7)	224 (30.9)	246 (49.7)	627 (53.9)
**By item**				
Item 1 correct (total calories)	107 (39.4)	599 (73.0)	518 (88.1)	968 (83.2)
Item 2 correct (nutrient-specific)	136 (53.9)	640 (75.3)	536 (87.5)	1,005 (87.6)
Item 3 correct (health recommendation)	56 (25.3)	403 (52.3)	382 (66.3)	809 (69.8)
Item 4 correct (daily value)	52 (18.8)	359 (48.5)	371 (72.7)	857 (74.7)

a Nutrition label scores were the total number of correct answers out of 4 Nutrition Facts panel questions (range: 0–4). Scores were based on answers to the following questions: Question 1: “If you eat the entire container, how many calories will you eat?”; question 2, “If you are allowed to eat 60 g of carbohydrates as a snack, how much ice cream could you have?”; question 3, “Your doctor advises you to reduce the amount of saturated fat in your diet. You usually have 42 g of saturated fat each day, which includes 1 serving of ice cream. If you stop eating ice cream, how many grams of saturated fat would you be consuming each day?”; question 4, “If you usually eat 2,500 calories in a day, what percentage of your daily value of calories will you be eating if you eat one serving?” Nutrition Label Score frequencies do not total to 3,165 because of missing data on education.

The first 2 NFP label questions (Total Calories and Nutrient Specific) were correctly answered by only 39.4% and 53.9% of people without a high school diploma, respectively. Questions 3 and 4 (Health Recommendation and Daily Value) were correctly answered by an even lower proportion of those without a high school diploma (25.3% and 18.8%, respectively). Across all educational levels, participants had the greatest difficulty with these last 2 questions (Table 2).

### NFP label understanding and self-reported dietary behaviors

In unadjusted models, NFP label understanding was negatively associated with sugar-sweetened soda consumption: participants with a better understanding of the NFP reported consuming sugar-sweetened soda fewer days per week (odds ratio [OR] = 0.88; 95% confidence interval [CI], 0.81–0.94; *P* = .001). NFP label understanding was positively associated with vegetable consumption (OR = 1.12; 95% CI, 1.03–1.21; *P* = .009) and positively associated with fruit consumption, although the latter was not significant (OR = 1.08; 95% CI, 1.00–1.17; *P* = .055) (Table 3).

**Table 3:** Self-Reported Dietary Behaviors, by Nutrition Facts Panel Scores^a^, Health Information National Trends Survey, United States, 2013

Behavior	Unweighted No. (Weighted %)	Nutrition Label Score, Mean (95% CI)^a^	OR (95% CI)^b^	*P* Value
**Soda consumption, days/week**				
Never	1,290 (36.8)	2.90 (2.80–3.00)	0.88 (0.81–0.94)	.001
<1	745 (23.1)	2.72 (2.55–2.90)		
1 or 2	498 (15.9)	2.73 (2.54–2.92)		
3 or 4	233 (8.6)	2.38 (1.99–2.76)		
5 or 6	91 (3.7)	2.56 (2.14–2.98)		
Every day	270 (11.8)	2.57 (2.27–2.88)		
**Vegetable consumption, cups/day**				
None	154 (4.7)	1.96 (1.57–2.35)	1.12 (1.03–1.21)	.009
≤½	480 (16.7)	2.57 (2.34–2.80)		
½ –1	747 (23.7)	2.89 (2.75–3.03)		
1–2	973 (31.4)	2.78 (2.66–2.89)		
2–3	463 (14.4)	2.79 (2.60–2.97)		
3–4	179 (6.1)	3.02 (2.74–3.29)		
>4	103 (2.9)	2.61 (2.27–2.95)		
**Fruit consumption, cups/day**				
None	242 (6.9)	2.19 (1.88–2.50)	1.08 (1.00–1.17)	.055
≤½	584 (20.3)	2.77 (2.60–2.95)		
½–1	769 (25.0)	2.75 (2.61–2.90)		
1–2	902 (29.6)	2.76 (2.61–2.91)		
2–3	374 (11.5)	2.84 (2.65–3.04)		
3–4	154 (4.7)	3.02 (2.64–3.39)		
>4	73 (2.1)	2.39 (1.83–2.95)		
**Total**	3,165 (100.0)	2.72 (2.65–2.79)	—	—

Abbreviations: —, not applicable; CI, confidence interval; OR, odds ratio.

a Nutrition label scores were the total number of correct answers out of 4 Nutrition Facts panel questions (range: 0–4). Scores were based on answers to the following questions: Question 1: “If you eat the entire container, how many calories will you eat?”; question 2, “If you are allowed to eat 60 g of carbohydrates as a snack, how much ice cream could you have?”; question 3, “Your doctor advises you to reduce the amount of saturated fat in your diet. You usually have 42 g of saturated fat each day, which includes 1 serving of ice cream. If you stop eating ice cream, how many grams of saturated fat would you be consuming each day?”; question 4, “If you usually eat 2,500 calories in a day, what percentage of your daily value of calories will you be eating if you eat one serving?” Unweighted frequencies do not total to 3,165 because of missing data on dietary behaviors.

b Odds ratios are from weighted simple ordinal logistic regressions of dietary behaviors on nutrition label scores.

After adjusting for demographic characteristics, the association between NFP label understanding and sugar-sweetened soda consumption remained significant (Table 4). Other significant predictors of sugar-sweetened soda consumption included sex, education level, age, and race/ethnicity, with higher soda consumption reported by people who were male, less educated, younger, and non-Hispanic black (Table 4).

**Table 4:** Self-Reported Dietary Behaviors, by Demographic Factors and Nutrition Facts Panel Scores^a^, Health Information National Trends Survey, United States, 2013

Characteristic	Sugar-Sweetened Soda Consumption		Vegetable Consumption		Fruit Consumption	
	OR (95% CI)^b^	*P* Value	OR (95% CI)^b^	*P* Value	OR (95% CI)^b^	*P* Value
**Sex**						
Female	1 [Reference]	—	1 [Reference]	—	1 [Reference]	—
Male	1.72 (1.33–2.23)	<.001	0.74 (0.59–0.92)	.009	0.82 (0.64–1.03)	.09
**Education**						
<High school diploma	2.58 (1.63–4.10)	<.001	0.52 (0.29–0.93)	.03	0.51 (0.32–0.82)	.006
High school diploma	2.03 (1.43–2.89)	<.001	0.68 (0.48–0.95)	.02	0.63 (0.47–0.84)	.003
Some college	1.71 (1.28–2.29)	.001	0.82 (0.58–1.16)	.26	0.76 (0.54–1.06)	.11
4-year college degree	1 [Reference]	—	1 [Reference]	—	1 [Reference]	—
**Income, $**						
<20,000	1.24 (0.79–1.93)	.35	0.88 (0.58–1.33)	.53	0.72 (0.54–0.98)	.03
20,000–<35,000	1.82 (1.13–2.94)	.02	0.87 (0.61–1.24)	.43	0.63 (0.43–0.93)	.02
35,000–<50,000	0.93 (0.57–1.51)	.75	0.69 (0.44–1.10)	.12	0.77 (0.51–1.18)	.23
50,000–<75,000	1.23 (0.81–1.87)	.32	0.90 (0.64–1.26)	.53	0.83 (0.58–1.19)	.31
≥$75,000	1 [Reference]	—	1 [Reference]	—	1 [Reference]	—
**Age, y**						
18–34	6.84 (4.33–10.80)	<.001	0.66 (0.42–1.04)	.08	0.66 (0.47–0.93)	.02
35–49	3.53 (2.36–5.27)	<.001	0.85 (0.58–1.24)	.39	0.77 (0.54–1.09)	.14
50–64	2.28 (1.53–3.41)	<.001	0.89 (0.64–1.24)	.49	0.71 (0.51–0.99)	.046
65–74	1.71 (1.01–2.90)	.047	0.86 (0.58–1.28)	.46	0.78 (0.54–1.12)	.17
≥75	1 [Reference]	—	1 [Reference]	—	1 [Reference]	—
**Race/ethnicity**						
White	1 [Reference]	—	1 [Reference]	—	1 [Reference]	—
Non-Hispanic black	1.46 (1.03–2.09)	.04	0.67 (0.39–1.13)	.13	1.00 (0.71–1.42)	.98
Hispanic	1.05 (0.73–1.52)	.78	0.77 (0.52–1.16)	.20	1.23 (0.86–1.75)	.26
Other	0.79 (0.55–1.13)	.19	1.75 (0.81–3.76)	.15	1.24 (0.79–1.97)	.34
**Nutrition Label Score**	0.90 (0.81–0.99)	.03	1.05 (0.92–1.21)	.45	1.03 (0.93–1.14)	.59

Abbreviations: —, not applicable; CI, confidence interval; OR, odds ratio.

a Nutrition label scores were the total number of correct answers out of 4 Nutrition Facts panel questions (range: 0–4). Scores were based on answers to the following questions: Question 1: “If you eat the entire container, how many calories will you eat?”; question 2, “If you are allowed to eat 60 g of carbohydrates as a snack, how much ice cream could you have?”; question 3, “Your doctor advises you to reduce the amount of saturated fat in your diet. You usually have 42 g of saturated fat each day, which includes 1 serving of ice cream. If you stop eating ice cream, how many grams of saturated fat would you be consuming each day?”; question 4, “If you usually eat 2,500 calories in a day, what percentage of your daily value of calories will you be eating if you eat one serving?”

b Odds ratios are from weighted ordinal logistic regressions in which all predictor variables were entered simultaneously.

The associations between NFP label understanding and fruit and vegetable consumption were not significant in models adjusted for demographic factors (Table 4). Female participants reported eating more vegetables than did male participants, those with a 4-year college degree reported eating more fruits and vegetables than those with a high school diploma or less, those in the highest income bracket reported eating more fruit than those in the 2 lowest income brackets, and those in the oldest age group reported eating more fruit than those in the youngest age group and the 50 to 64-year age group (Table 4).

## Discussion

We assessed understanding of an NFP label among a representative sample of US adults and examined associations among label understanding, self-reported dietary behaviors, and sociodemographic characteristics. Although other studies — which have included convenience samples of patients, consumers, parents, and children — have examined how well people interpret information on a nutrition label (6,10,12), ours is one of the few studies to examine understanding of nutrition labels by using nationally representative data. The National Health and Nutrition Examination Survey, which assesses the health and nutrition of US children and adults, has surveyed whether people use the NFP label but not whether they can actually apply the information contained on the label (21). We examined critical aspects of nutrition label understanding: whether people could calculate the total number of calories in an entire container of ice cream, the number of servings of ice cream equal to 60 g of carbohydrates, the effect that foregoing a serving of ice cream would have on saturated fat intake, and the percentage daily value of calories represented by one serving of ice cream. Given our large sample, we were able to examine the association between nutrition label understanding and 3 dietary behaviors.

In general, participants’ ability to interpret nutrition label information was poor. Understanding was lower among older than younger adults, Hispanic and non-Hispanic black people than non-Hispanic white people, lower income than higher income people, and less educated than more educated people. People who had not graduated from high school had the poorest performance on the 4 nutrition label questions: more than a third of this group could not correctly answer any nutrition label questions, and fewer than 9% could correctly answer all 4 questions. These findings are consistent with those of other studies that have found low educational attainment to be associated with poor understanding of nutrition labels (10,22).

Even a college education did not ensure nutrition label understanding. Only slightly more than half (53.9%) of people who had earned a 4-year college degree could correctly answer all 4 nutrition label questions. Other studies of college students have reported similar difficulties interpreting nutrition labels. In one study of over 500 undergraduate and graduate students, nearly 90% of students could not define serving size, and one-third could not perform simple tasks involving comparison of nutrition labels (9). Another college student survey found that only 60% of the approximately 200 students surveyed could use the information on a nutrition label to determine the number of servings of a particular food that would meet 100% of the daily value of carbohydrates for a 2,000-calorie diet (23).

Nutrition label understanding was also associated with dietary behaviors: compared with people with lower levels of nutrition label understanding, those with better label understanding tended to eat more vegetables and drink less sugar-sweetened soda. These findings are consistent with those of another study that found that healthier dietary practices were positively associated with greater nutrition label understanding (24).

The finding that only slightly more than half of people with a college degree could correctly answer all 4 nutrition label questions — each of which involved a single-step arithmetic operation — underscores the importance of health literacy. Using NFP labels requires not only being able to read and perform arithmetic but also — just as importantly — the ability to reason with words and numbers. According to our results, a substantial proportion of consumers clearly struggle to effectively use the information contained in a nutrition label. Across all education levels, participants performed best on Item 1 (Total Calories) and Item 2 (Nutrient Specific). We can only speculate as to why Items 3 (Health Recommendation) and Item 4 (Daily Value) proved more difficult. It is possible that these items required more complex reasoning than the other items. Item 3 was particularly long, comprising 3 sentences, and Item 4 required participants to understand percentages, which may have presented a challenge.

Nutrition labels can be a useful source of information for managing one’s diet and diet-related health conditions (25), and simple, clear nutrition labels are critical to ensuring that everyone can benefit from dietary information. The revised NFP label, which the US Food and Drug Administration (FDA) updated in 2016 to reflect current nutrition science and public health research, attempts to make the nutrition label more user-friendly. Major changes to the label included making certain label elements more salient (eg, calories, servings per container, and serving size are highlighted by a larger font size and bold type), updating serving sizes to more accurately reflect the amount of food and drink that people usually consume, and updating nutrient daily values to be consistent with the Dietary Guidelines for Americans (26). Sugar content now includes added sugars in grams and as a percentage of daily value. To help consumers better understand serving size, dual column labels will be used for foods that can be eaten in one or multiple sittings (eg, a bag of potato chips), so people will be able to easily determine how many calories and nutrients they consume if they eat the package contents in one sitting (26).

On the basis of responses to NVS questions, the revised nutrition label may make it easier for consumers to understand the total calories per food item (Item 1, Total Calories ) and to make better use of specific nutrient information (Item 2, Nutrient Specific). However, the label revisions would not necessarily improve people’s ability to understand the effect that foregoing a serving of a food item would have on their nutrient intake (Item 3, Health Recommendation) or to calculate the percentage of their daily value of calories represented by one serving of a food item (Item 4, Daily Value). The latter tasks still require consumers to perform calculations and interpret those calculations. 

As nutrition labels are improved to make them easier for all consumers to use, schools also have a role to play in teaching students the skills they need to understand the labels and make informed dietary decisions. This education could be achieved by integrating nutrition education and STEM (science, technology, engineering, and math) across all school levels (elementary through high school). Additionally, states could adopt school nutrition education policies requiring a standards-based health education curriculum integrating nutrition education content with specific standards across grade levels. As of December 31, 2014, few states had such strong state nutrition education policies; 7 states had strong policies for elementary schools and 6 states had strong polices at the middle and high school levels (https://class.cancer.gov/map_nutrition.aspx).

Our study has limitations. Because study data were collected by mail survey, participants who had difficulty answering the NFP label questions may have obtained help from a family member or other source. If this were the case, we may have overestimated NFP label understanding. The mail survey may also have excluded people with low literacy, which would have further inflated our estimates of NFP label understanding. Space limitations on the HINTS survey instrument precluded including all 6 nutrition label questions originally developed as part of the NVS. Including these 2 additional questions may have enhanced the reliability of our estimate of NFP label understanding. Moreover, because dietary behaviors were self-reported, the data may have been subject to reporting bias, social desirability bias, or random error (27,28). The validity of memory-based dietary recall is an ongoing debate in nutrition research (28,29). It may have been challenging for participants to estimate the number of cups of fruits and vegetables they typically eat, even though the survey instrument provided examples of portion size.

Additional research on health literacy in the context of nutrition should also consider the emerging literature on nutrition and food literacy (30), concepts developed to broaden the scope of research and practice in this area. Like other studies (30), our study focused solely on functional nutrition literacy and did not include other aspects of nutrition literacy, such as interactive or critical nutrition literacy (30). For example, understanding NFP labels is not the same as using the nutritional information on the label for selecting food. That is, participants who answered all 4 nutrition label questions correctly may not necessarily use these labels when purchasing food (an example of interactive nutrition literacy) (30).

A substantial proportion of consumers in this country, including those with a college education, have difficulty understanding NFP labels, which is likely a function of limited health literacy. This deficit would be expected to limit consumers’ ability to apply the information contained in a nutrition label effectively and compromise their ability to make informed dietary choices. Future research should examine whether the revised NFP label promotes greater understanding, healthier dietary choices, and ultimately, better health outcomes.
